# Analysis of the independent risk factors of neurologic deficit after thoracolumbar burst fracture

**DOI:** 10.1186/s13018-016-0448-0

**Published:** 2016-10-24

**Authors:** Peifu Tang, Anhua Long, Tao Shi, Licheng Zhang, Lihai Zhang

**Affiliations:** Department of Orthopedics, General Hospital of Chinese PLA, No. 28 Fuxing Road, Beijing, 100853 People’s Republic of China

**Keywords:** Spinal fractures, Spinal cord compression, Neurological examination, Computed tomography, Logistic models

## Abstract

**Background:**

The objective of this study is to identify the independent risk factors of neurologic deficit after thoracolumbar burst fracture. Traumatic fractures of the thoracolumbar spine are the most common type of spinal column fractures. Many studies have attempted to determine whether neurologic deficit in such fractures is related to spinal canal stenosis or other parameters observed on axial computed tomography. However, this relationship remains controversial.

**Methods:**

A review of the clinical data and axial computed tomography (CT) for 105 patients was performed. Neurologic deficit was classified according to the American Spinal Injury Association (ASIA) classification. Various preoperative CT parameters, including vertebral body compression, canal stenosis, sagittal alignment, and fragment reverse, were analyzed using ordinal logistic regression analysis.

**Results:**

*Arbeitsgemeinschaft für Osteosynthesefragen* (AO) classification, canal volume, transverse canal diameter, median sagittal diameter, Cobb angle, compression ratio of the sagittal diameter, compression ratio of the cross-sectional area, and compression ratios of the anterior vertebral height (AVH), middle vertebral height (MVH), and posterior vertebral height (PVH) were significantly associated with severity of nerve injury (*P* < 0.05). However, flip angle and rotation angle of bony fragments were unrelated to severity of nerve damage. Multivariate logistic regression identified AO classification, compression ratio of median sagittal diameter, anterior vertebral compression ratio, and distance from the posterior margin to the vertebral body above to be independent variables associated with neurologic deficit.

**Conclusions:**

The four CT parameters most strongly associated with neurologic deficit in thoracolumbar burst fractures are AO classification, compression ratio of median sagittal diameter, anterior vertebral compression ratio, and distance from the posterior margin to the vertebral body above.

## Background

Traumatic fractures of the thoracolumbar spine, especially of the thoracolumbar junction (T10-L2), are the most common spinal column fractures. High activity and lack of stability make the thoracolumbar spine more prone to fracture. Thoracolumbar burst fracture, defined as a fracture or comminution of both the anterior and middle columns with retropulsion of bony fragments into the spinal canal [1], accounts for approximately 50 to 60 % of all thoracolumbar fractures that cause neurologic deficit [2, 3].

Many studies have attempted to determine whether neurologic deficit in such fractures is related to spinal canal stenosis or other parameters observed on axial computed tomography (CT). However, this relationship remains controversial [3–10]. In 1992, Fontijne et al. reported a positive correlation between neurologic deficit and spinal canal stenosis in 139 patients with thoracolumbar burst fracture [4]. However, there was no predictive value for the severity of neurologic deficit. Other imaging parameters have been shown in several other studies to be useful in predicting the severity of neurologic deficit after thoracolumbar burst fracture [11, 12]. In contrast, in 2008, Mohanty et al. found no association between the extent of canal stenosis and the severity of neurologic deficit [9]. Moreover, they found a significant correlation between thoracic spine, rather than L1, and the severity of neurologic deficit.

Most studies have emphasized that spinal canal stenosis and posterior ligamentous complex injury after a fracture lead to neurologic damage [13–16]. However, few reports shed light on the correlation between sagittal alignment, rotation angle of bony fragments, and severity of nerve damage, and no multivariate statistical analysis has considered all prospective indicators related to neurologic damage or found ways to assess neurologic deficit using radiographic parameters. In this study, four radiographic parameters were chosen to assess neurologic deficit after thoracolumbar burst fracture: vertebral body compression, canal stenosis, sagittal alignment, and reverse fragments. The purpose of this study was to identify independent risk factors correlating with neurologic deficit after thoracolumbar burst fracture using a multivariate logistic regression model. Through this study, we would like to analyze some parameters from CT scan that are strongly associated with neurologic deficit, in order to judge prognosis and guide surgery.

## Methods

### Patients

We reviewed the clinical records of patients admitted to our department from January 2009 to December 2011 with trauma-associated, single-segment thoracolumbar burst fracture without posterior longitudinal ligament damage. The inclusion criterion was trauma-associated, single-segment thoracolumbar burst fracture. The exclusion criteria were multi-level vertebral fractures, osteoporosis, cancer metastasis, spondylolisthesis, burst fracture with displacement, ankylosing spondylitis, and degenerative arthritis of the hip or knee. All investigations were carried out in accordance with the ethical guidelines and were approved by the Institutional Ethical Review Committee of Chinese PLA General Hospital (20090121).

### Experimental instruments

**Fig. 1:**
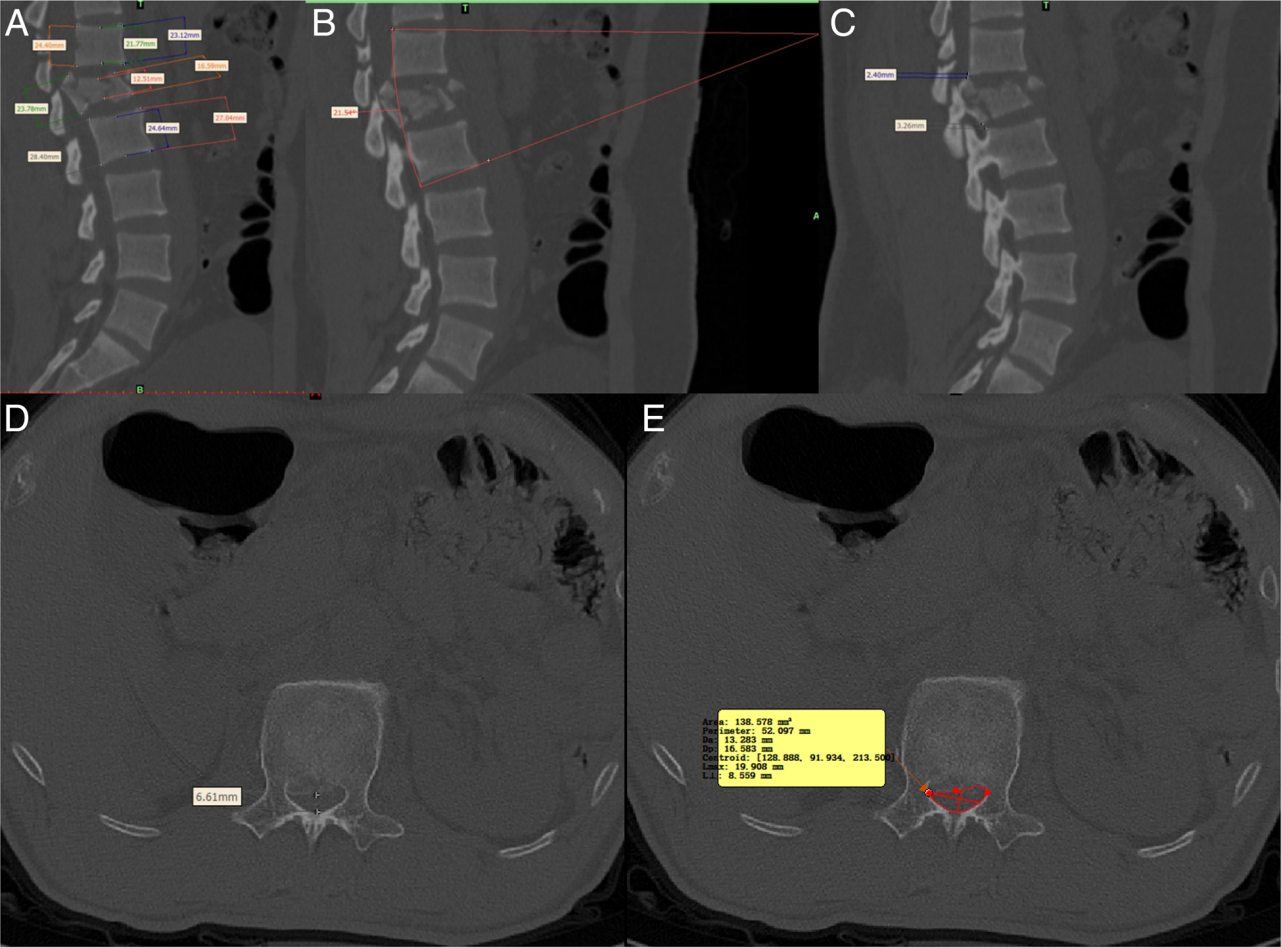
**a**: Anterior, median, and posterior vertebral wall heights. **b** Local kyphosis angle. **c** Distances of the posterior vertebral edge of the injured vertebrae from the upper and lower adjacent posterior vertebral walls. **d** Median vertebral canal sagittal diameter. **e** Cross-sectional area of the vertebral canal

A Somatom Sensation Open 40-slice CT scanner (Siemens AG, Erlangen, Germany) was used for preoperative CT scanning. The imaging workstation was from Siemens AG. syngo image-processing software (Siemens AG) was used for image analysis (Fig. 1).

### Measurement of imaging parameters

- □ *Vertebral body compression.* The average of anterior vertebral height, middle vertebral height, and posterior vertebral height (AVH, MVH, and PVH, respectively) of the vertebral bodies. From top to bottom of the injured vertebra was considered normal height. Compression ratios of AVH, MVH, and PVH were calculated [17].
- □ *Canal stenosis.* Median sagittal diameter and transverse canal diameter have been used as important indicators for a patient’s spinal condition. Thus, in the CT images of the pedicle level, those fracture fragments were obviously visible; the sagittal and transverse canal diameters of the spinal canal were measured.
  - ○ *Compression ratio of median sagittal diameter*. The compression ratio of the median sagittal diameter was calculated using an equation *a* = (1 – *x*/*y*) × 100 %. A means compression ratio of median sagittal diameter, where *x* means median sagittal diameter of the spinal canal at the fracture level and *y* means normal median sagittal diameter of the spinal canal equal to the average vertebral canal sagittal diameter of the segments from top to bottom [5].
  - ○ *Compression ratio of canal cross-sectional area.* When assessing the degree of compression of the spinal canal, compression ratio of the canal cross-sectional area was measured to assess spinal compression after sudden fracture. Average canal cross-sectional area of the segments from top to bottom was considered normal canal cross-sectional area [18].
- □ *Sagittal alignment.*
  - ○ *Distance from the posterior margin of fractured segment to that of the vertebral body from top to bottom.* The distance between the posterior margin of the fractured segment and that of the adjacent segment reflected the length of the posterior longitudinal ligament, which further reflected the injury of the spinal sequence [19].
  - ○ *Cobb angle.* The angle between a line parallel to the superior endplate of the vertebra above the fracture and a line parallel to the inferior endplate of the vertebra below the fracture reportedly has the highest reliability within and between groups [20].
- □ *Fragment rotation.*
  - ○ *Flip angle of bony fragment in sagittal plane*. In the sagittal plane, the flip angle was defined as the angle between a line parallel to the posterior wall of the bony fragment and that of a normal vertebral body from top to bottom [21].
  - ○ *Rotation angle of bony fragments in the horizontal plane.* In the horizontal plane, rotation angle was defined as the angle between a line parallel to the posterior wall of the bony fragment and that of a normal vertebral body from top to bottom [22, 23].
- □ *Arbeitsgemeinschaft für Osteosynthesefragen (AO) classification.* The Müller AO classification of fractures was used [24]. A3.1 Incomplete burst fracture: a fracture on the edge of a vertebral body of which the upper and lower endplates are basically intact and there is no intervertebral disc lesion. This type of fracture is given a score of 1 point. A3.2 Burst split fracture: a vertebral body split fracture with superior and inferior endplate lesions, with no intervertebral disc lesion (2 points). A3.3 Complete burst fracture: comminuted fracture of the vertebral body. Complete shattering of upper and lower endplates and no intervertebral disc lesion (2 points).

### Assessing neurologic deficit

Neurologic deficit was classified according to the American Spinal Injury Association (ASIA) classification [25].

### Statistical analysis

SPSS for Windows, Version 13.0 (SPSS Inc., Chicago, IL, USA), was used for statistical analysis. Normal distributions of continuous-variable data are reported as arithmetic mean ± standard deviation and non-normal distribution data as median (25th and 75th percentiles). A single-factor ordinal polytomous logistic regression analysis was used to compare differences in parameters between different ASIA grades. All possible parameters underwent multivariate, ordinal, polytomous logistic regression analysis. *P* < 0.05 was defined as statistically significant.

## Results

One hundred and thirty-seven patients with thoracolumbar burst fracture were admitted to our service from January 2009 to December 2011, of whom 105 patients (66 were male [62.8 %]; mean age 38.0 ± 11.8 years [range, 18–63 years]) met the inclusion criteria and 31 were excluded. Demographic information, including fracture levels and AO and ASIA grade, of the enrolled patients is shown in Table 1. Twenty AO type A patients (47.6 %), 15 AO type B patients (62.5 %), and 32 AO type C patients (82.1 %) suffered from neurologic deficit. There was a significant positive correlation between AO classification and severity of nerve injury (*P* = 0.001).

**Table 1:** Demographics of the thoracolumbar burst fracture patients included in this study

	Numbers	SD/%
Mean age	38.0	11.8
Male	66	62.8 %
Female	39	37.2 %
T11	10	9.5 %
T12	21	20.0 %
L1	45	42.9 %
L2	29	27.6 %
AO type A	42	40.0 %
AO type B	24	22.9 %
AO type C	39	37.1 %
ASIA A	38	36.2 %
ASIA B	17	16.2 %
ASIA C	14	13.3 %
ASIA D	15	14.3 %
ASIA E	21	20.0 %

Table 2 shows that CT parameters such as canal volume, transverse canal diameter, median sagittal diameter, compression ratio of sagittal diameter, and cross-sectional area were significantly associated with severity of nerve injury (*P* < 0.05). In other words, the smaller the transverse canal diameter or sagittal diameter, the more serious the nerve injury; the higher the compression ratio of sagittal diameter and cross-sectional area, the more serious the nerve damage. Compression ratios of the anterior, middle, and posterior vertebral heights were also positively correlated with ASIA grade. Thus, there was a significant likelihood of spinal cord compression and nerve damage if compression ratios were high (*P* = 0.001). With regard to sagittal alignment, the smaller the posterior margin distance, the greater the likelihood of nerve damage. However, no significant correlation was observed between the parameters of posterior margin distance and ASIA grade. Cobb angle and severity of nerve injury were positively correlated (*P* = 0.039). Flip angle and rotation angle of bony fragments were unrelated to severity of nerve damage (Table 2).

**Table 2:** Comparison between computerized tomographic parameters and ASIA classification

	ASIA A 38 cases		ASIA B 17 cases		ASIA C 14 cases		ASIA D 15 cases		ASIA E 21 cases		*P* value
AO classification	22	58 %	8	47 %	4	29 %	4	27 %	4	19.0 %	0.001
Transverse canal diameter	25.48	(22.65, 27.63)	26.54	(23.84, 28.16)	26.24	(23.44, 28.22)	24.19	(20.64, 28.82)	22.68	(0.01, 27.65)	0.003
Median sagittal diameter	9.76	(7.47, 13.94)	11.02	(7.29, 15.27)	12.03	(7.25, 14.01)	8.95	(6.17, 11.66)	6.27	(0.01, 8.76)	0.001
Compression ratio of median sagittal diameter	0.365	(0.07, 0.502)	0.308	(0.031, 0.504)	0.367	(0.084, 0.574)	0.476	(0.288, 0.788)	0.600	(0.434, 1.000)	0.001
Compression ratio of canal cross-sectional area	0.256	(0.045, 0.435)	0.274	(0.082, 0.445)	0.171	(0.046, 0.565)	0.218	(0.106, 0.44)	0.386	(0.246, 0.839)	0.004
Compression ratios of AVH	0.146	(0.107, 0.294)	0.190	(0.138, 0.276)	0.259	(0.147, 0.309)	0.386	(0.254, 0.47)	0.363	(0.256, 0.454)	0.001
Compression ratios of MVH	0.104	(0.068, 0.185)	0.097	(0.056, 0.17)	0.158	(0.078, 0.225)	0.199	(0.075, 0.277)	0.248	(0.105, 0.343)	0.001
Compression ratios of PVH	0.191	(0.112, 0.281)	0.181	(0.107, 0.247)	0.259	(0.189, 0.341)	0.276	(0.119, 0.387)	0.286	(0.181, 0.392)	0.005
The distance of posterior margin with upper vertebra	4.95	(4.31, 6.12)	5.02	(3.64, 5.92)	4.76	(3.51, 5.86)	5.08	(4.02, 6.49)	4.65	(3.97, 5.15)	0.200
The distance of posterior margin with below vertebra	3.98	(3.27, 4.66)	4.29	(3.25, 5.24)	3.86	(3.21, 4.64)	3.59	(2.76, 4.23)	3.66	(3.36, 4.89)	0.302
Cobb angle	10.3	(8.6, 14.0)	11.3	(2.9, 21.0)	10.7	(7.1, 17.9)	12.9	(10, 18.7)	16.2	(9.4, 22.8)	0.039
Flip angle	16.44	(10.24, 31.59)	15.90	(7.92, 28.3)	32.26	(15.47, 43.6)	28.26	(10.9, 42.31)	21.61	(10.54, 37.64)	0.189
Rotation angle	0.10	(0.1, 4.75)	2.95	(0.1, 3.94)	3.21	(0.1, 5.8)	4.30	(1.96, 5.63)	2.52	(0.1, 5.46)	0.366

Table 3 presents the results of multivariate logistic regression analysis of AO classification and all parameters that may cause nerve damage. We found that AO fracture classification, compression ratio of median sagittal diameter, anterior vertebral compression ratio, and distance from the posterior margin of the fractured segment to that of the vertebral body above were four independent predictors of severity of nerve damage after thoracolumbar burst fracture.

**Table 3:** Multivariate analysis using ordinal logistic regression models to evaluate the independent risk factors of neurologic deficit after thoracolumbar burst fracture

	OR	95 % CI		*P* value
Transverse canal diameter	0.96	0.9	1.02	0.203
Median sagittal diameter	1.06	0.89	1.27	0.513
Compression ratio of median sagittal diameter	*26.1*	*1*	*684.71*	*0.050*
Compression ratio of canal cross-sectional area	0.94	0.05	16.59	0.966
Compression ratios of AVH	*533.79*	*10.09*	*28226.03*	*0.002*
Compression ratios of MVH	27.03	0.2	3662.86	0.188
Compression ratios of PVH	0.01	0	3.03	0.118
The distance of posterior margin with upper vertebra	*0.62*	*0.43*	*0.9*	*0.013*
The distance of posterior margin with below vertebra	1.08	0.72	1.63	0.712
Cobb angle	1.01	0.94	1.06	0.952
Flip angle	0.98	0.95	1.01	0.091
Rotation angle	1.03	0.99	1.07	0.196
AO classification type A	*0.18*	*0.06*	*0.54*	*0.002*
AO classification type B	*0.31*	*0.1*	*0.96*	*0.042*

## Discussion

Few reports shed light on the correlation between sagittal alignment, rotation angle of bony fragments, and severity of nerve damage, and no multivariate statistical analysis has considered all prospective indicators related to neurologic damage or found ways to assess neurologic deficit using radiographic parameters. In this study, four radiographic parameters were chosen to assess neurologic deficit after thoracolumbar burst fracture: vertebral body compression, canal stenosis, sagittal alignment, and reverse fragments. The purpose of this study was to identify independent risk factors correlating with neurologic deficit after thoracolumbar burst fracture using a multivariate logistic regression model.

Denis investigated 412 patients with spinal fractures and proposed the spinal three-column theory to create a more detailed discussion of fractures using CT images. As defined using this theory, bony fragments from thoracolumbar burst fractures are retropulsed with greater intensity into the spinal canal.

Many studies focus on the complications including neurological status after thoracolumbar fractures [13–16]. Andreas reported preoperative neurological deficits (American Spinal Injury Association (ASIS) Classification), 15 ASIS B deficits and 8 ASIS C deficits in 25 patients with thoracolumbar fractures (T11-L2), with Denis classifications. Palvos reported preoperative neurological deficits (ASIS classification), 75 ASIS E deficits and 25 ASIS (A–D) deficits in 100 patients of thoracolumbar fractures (T11-L2), with AO-Magerl classification. Kalliopi discussed and reported that integrity of the posterior ligament has some relationship with neurological deficits. However, all the discussions focus on the main classification and not on the specific parameters.

Our results indicate that four radiographic parameters correspond to the severity of nerve damage after thoracolumbar burst fracture: AO classification of fracture, compression ratio of median sagittal diameter, anterior vertebral compression ratio, and the distance from the posterior margin to that of the vertebral body above. Most previous studies considered canal stenosis after spinal fracture to be the main indicator of nerve damage. Spinal cord compression was assessed by measuring the sagittal diameter of the spinal canal on axial CT. Some studies consider canal volume to reflect nerve damage, but this is still controversial. Rasmussen et al. [15] found that the cross-sectional area of the vertebral canal was a better parameter by which to assess spinal compression. Compared with compression ratio of sagittal diameter, the compression ratio of cross-sectional area had a stronger correlation with neurological function. However, cross-sectional area of the spinal canal varies with sex and ethnicity. When assessing compression of the spinal canal after fracture, use of the compression ratio of the cross-sectional area may effectively avoid bias associated with sex and ethnicity. In our study, compression ratio of sagittal diameter and cross-sectional area were associated with nerve injury. However, multivariate analysis showed that compression ratio of sagittal diameter had a stronger correlation with the severity of nerve damage than did the compression ratio of the cross-sectional area.

Rotation of retropulsion bony fragments in the vertebral canal as well as change in canal volume was taken into account. Thoracolumbar burst fractures create bony fragments of the posterior wall, which are retropulsed into the vertebral canal. Guerra et al. [26] found retropulsion and rotation of bony fragments, a phenomenon that was later confirmed on CT. Flipping of bone fractures may reflect the severity of injury and may therefore predict nerve damage. However, according to our study, flip angle and rotation angle of bony fragments were unrelated to severity of nerve damage.

Changes in vertebral height may reflect severity of canal stenosis and nerve compression. Parameters including the compression ratios of the AVH, MVH, and PVH; vertebral flip angle; and cross-diagonal angle are widely used to measure changes in vertebral height [11]. It is also generally believed that surgery may be indicated for a vertebral compression ratio of >50 % after thoracolumbar burst fracture [14]. AVH, MVH, and PVH compression ratios may accurately reflect the severity of nerve injury after fracture, with the strongest correlation being between the ratio of PVH and the severity of nerve injury.

Changes in the overall sequence of vertebrae often occur after thoracolumbar burst fracture, with disruption of the relative positions of the upper and lower vertebrae. Therefore, we measured both Cobb angle and the distance between the posterior margin of the fracture segment and the neighboring segment to assess changes in vertebral sequence. The results showed that refined Cobb angle was promising in its ability to predict the severity of the fracture and nerve damage. Cobb angle was originally used to describe the integrity of the posterior longitudinal ligament and deformity in the coronal plane. It was also used to assess degree of spinal vertebral kyphosis [27]. However, no report to date has shown a relationship between Cobb angle and nerve injury after thoracolumbar burst fracture.

A number of previous studies considered canal volume as well as vertebral height to be predictors of nerve injury after thoracolumbar burst fracture [4, 7–10]. In the present study, we performed statistical analysis of radiographic parameters of axial CT, such as compression of canal cross-sectional area and vertebral height, to find the parameters most closely associated with nerve damage and found spinal canal volume, vertebral height, and sagittal alignment to be the best predictors. AO classification was also a good predictor of the degree of nerve damage.

There are some limitations in this study. Firstly, all the measurements were made by human beings; there must be some measurement bias. Additionally, the patient’s number is limited because of the number of fractures. Lastly, the CT scan can only reflect the situation when the patient did the test; the mechanism of injury and the injury moment of fracture should be considered. A multicenter study and more accuracy measurements or 3D measurements in the spinal canal will better the design for further studies.

## Conclusions

Our study indicated that the four CT parameters with the strongest associations with neurologic deficit than that of other parameters in thoracolumbar burst fractures were AO classification of fracture, compression ratio of median sagittal diameter, anterior vertebral compression ratio, and distance from the posterior margin to the vertebral body above.

## Abbreviations

AO: Arbeitsgemeinschaft für Osteosynthesefragen
ASIA: The American Spinal Injury Association
AVH: Anterior vertebral height
CT: Computed tomography
MVH: Middle vertebral height
PVH: Posterior vertebral height
